# Establishment of a five‐enzalutamide‐resistance‐related‐gene‐based classifier for recurrence‐free survival predicting of prostate cancer

**DOI:** 10.1111/jcmm.17554

**Published:** 2022-09-28

**Authors:** Jing Chen, Jialin Meng, Yi Liu, Zichen Bian, Qingsong Niu, Junyi Chen, Jun Zhou, Li Zhang, Meng Zhang, Chaozhao Liang

**Affiliations:** ^1^ Department of Urology The First Affiliated Hospital of Anhui Medical University Institute of Urology and Anhui Province Key Laboratory of Genitourinary Diseases Anhui Medical University Hefei Anhui China; ^2^ Urology Institute of Shenzhen University The Third Affiliated Hospital of Shenzhen University Shenzhen University Shenzhen China

**Keywords:** classifier, enzalutamide, prostate cancer, recurrence‐free survival

## Abstract

To identify prostate cancer (PCa) patients with a high risk of recurrence is critical before delivering adjuvant treatment. We developed a classifier based on the Enzalutamide treatment resistance‐related genes to assist the currently available staging system in predicting the recurrence‐free survival (RFS) prognosis of PCa patients. We overlapped the DEGs from two datasets to obtain a more convincing Enzalutamide‐resistance‐related‐gene (ERRG) cluster. The five‐ERRG‐based classifier obtained good predictive values in both the training and validation cohorts. The classifier precisely predicted RFS of patients in four cohorts, independent of patient age, pathological tumour stage, Gleason score and PSA levels. The classifier and the clinicopathological factors were combined to construct a nomogram, which had an increased predictive accuracy than that of each variable alone. Besides, we also compared the differences between high‐ and low‐risk subgroups and found their differences were enriched in cancer progression‐related pathways. The five‐ERRG‐based classifier is a practical and reliable predictor, which adds value to the existing staging system for predicting the RFS prognosis of PCa after radical prostatectomy, enabling physicians to make more informed treatment decisions concerning adjuvant therapy.

## 1 BACKGROUND

As one of the most malignant tumours in males of the world, prostate cancer (PCa) has approximately 1,00,000 diagnosed cases diagnosed every year.^1^ At present, PCa has developed into the second leading cause of cancer‐related deaths in males in developed countries. Genetic and demographic factors, such as race, age and family history, are closely related to the occurrence and progression of PCa.^2^, ^3^ As our understanding of the underlying biology of PCa has gradually broadened, various treatment strategies have been developed, such as radical prostatectomy, androgen deprivation therapy (ADT), radiation therapy and chemotherapy. However, the prognosis of PCa is still unsatisfactory, and most tumours recur to castration resistance within two years.^4^

In 1941, Huggins and Hodges discovered that ADT can be the effective treatment to improve the prognosis of PCa.^5^ Since then, ADT has been regarded as the basis of advanced PCa management. According to the survey, about half of prostate cancer patients will receive ADT for a period of time during treatment in the United States.^5^, ^6^ Although tumour regression can be caused by ADT through inhibiting the androgen signalling in most cases, it also will inevitably develop into castration resistance that the tumour cells will lead to the progression of disease for adapting to low androgen levels.^7^, ^8^, ^9^ Potent anti‐androgens is one of the treatment options for metastatic castration‐resistant prostate cancer (mCRPC) through physical competition with the receptor's natural ligand dihydrotestosterone (DHT) to target the AR or inhibiting the biosynthesis of androgen.^10^ Currently, enzalutamide (MDV‐3100), as a prescribed compound, is most frequently used to treat mCRPC.^11^, ^12^, ^13^, ^14^, ^15^ As the direct androgen receptor inhibitor, enzalutamide ultimately eliminates the expression of androgen‐responsive genes by affecting the AR signalling pathway at multiple sites, such as preventing ligand binding, inhibiting AR nuclear translocation and blocking DNA transactivation.^16^, ^17^ The multi‐stage effect of enzalutamide on AR signal transduction is considered to be the main reason why its clinical activity is better than over other direct AR inhibitors, for example, flutamide, bicalutamide and nilutamide.^18^ However, the response of patients to the treatment of enzalutamide is different because of the heterogeneity between PCa patients.^19^ Currently, it is not easy to obtain RNA profiles from patients who received Enzalutamide treatment and progressed to drug resistance, which could provide resistance‐related gene markers, and served as prognostic markers for PCa patients.

Here, we employed microarray analysis to reveal the expression differences between Enzalutamide treatment resistant and parental cells. We also analysed the DEGs of a RNA‐sequencing study that focused on pre‐ and post‐Enzalutamide treatment based on patient‐derived Xenograft (PDX) models. Subsequently, we correlated the differentially expressed genes (DEGs) overlapped from the two cohorts with recurrence‐free survival (RFS) of PCa patients and established the RFS predicting classifier using LASSO Cox regression analysis. We validated the classifier in five independent cohorts, as well as our centre samples and obtained satisfying results. Besides, the functional and immunohistochemistry (IHC) assays proved the biological role and clinical significance of these critical candidates. Our findings added clinical values to the currently available prognostic predicting systems and facilitated the personalized treatment of PCa.

## 2 MATERIALS AND METHODS

### 2.1 Cell culture

Androgen‐independent cell line C4‐2 and 22Rv1 were purchased from American Type Culture Collection (ATCC, Manassas, VA). C4‐2 was used as an Enzalutamide‐sensitive cell line, and we developed an Enzatalumide treatment resistance cell line, C4‐2R by maintaining with high‐dose of Enzalutamide for at least 6 months, while 22Rv1 cells are naturally resistant to Enzalutamide. The C4‐2, C4‐2R and 22Rv1 cell lines were cultured in RPMI‐1640 (Gibco, Waltham, MA), which supplemented with 10% foetal bovine serum (Gibco, Waltham, MA), streptomycin (100 ng/ml) and penicillin (100 U/ml) (Gibco, Waltham, MA). For maintaining the Enzalutamide resistance, C4‐2R cells were cultured in RPMI‐1640 and supplemented with Enzalutamide (10 μM; TargelMol, Wellesley Hills, MA). Cells were incubated at 37°C with 5% CO_2_.

### 2.2 Microarray

Total RNA was extracted from the C4‐2 and C4‐2R cells. Through using the Agilent 2100 bioanalyzer and RNA LabChip kits, the quality and integrity of the RNA were evaluated. In line with the instructions of manufacturer (Shanghai OE Biotech. Co., Ltd.), the microarrays were generated by using 200 ng of total RNA. Microarrays were scanned by the Scanner C slide holder. The Feature Extraction software was used to generate the raw data after generating the microarray scan images. For assembling mapped reads from each sample, StringTie (version 1.3.1) was used that the reference sequence was refined as a guide. The Cuffdiff (version 2.1.1) was used to estimate the FPKM of the genes.^20^ Through using the Ballgown package,^21^ the expression levels of genes in different groups were compared. The cut‐off criteria was set to the absolute [log_2_ (fold change)] > 1.0 and the adjusted *p*‐value <0.05. If the expression of genes met the above rules, it was classified as differentially expressed.

### 2.3 Data collection

From UCSC Xena (https://tcga.xenah ubs.net), we downloaded the RNA‐seq and clinical information of TCGA‐PRAD. We also downloaded five datasets from the National Center For Biotechnology Information (NCBI), Gene Expression Omnibus (GEO, https://www.NCBI.nlm.nih.gov/geo/), including GSE70380, GSE116918, GSE70769, GSE46602 and GSE21032 (also termed as Memorial Sloan‐Kettering Cancer Center, MSKCC) datasets, fulfilling the following criteria: (1) PCa cases with available expression data; and (2) available information on clinicopathological features, particularly for RFS status and time.

### 2.4 Data processing

For TCGA‐PRAD cohort, firstly, fragments per kilobase of non‐overlapped exons per million fragments mapped (FPKM)’s number was calculated, following with that the FPKM was transferred into transcripts per kilobase million (TPM) values, which are similar to those resulting from microarrays and more comparable between samples, and then transferred to log_2_ (TPM + 1) value for downstream analysis. For the MSKCC cohort, the normalized log_2_ mRNA expression was download from http://cbio.mskcc.org/cancergenomics/prostate/data/. The gene matrix data of GSE70380, GSE116918, GSE70769 and GSE46602 were directly downloaded from the GEO database, and the expression of all genes was pre‐log_2_‐transferred. We also collected the preprocessed TPM data from our own AHMU‐PC cohort for the external validation, the obtain details and clinical information provided in our published work.^22^

### 2.5 Access to differentially expressed genes

For our RNA sequence count data, the ‘DESeq2’ was used to identify the DEGs between C4‐2R with C4‐2 cells. For the RNA‐seq data of GSE70380, the ‘DESeq2’ package was used to analyse the DEGs between pre‐ and post‐Enzalutamide treatment based on patient‐derived Xenograft (PDX) models. For ruling out false‐positives, the adjusted *P* value <0.05, false discovery rate (FDR) < 0.05 and absolute log_2_ (fold change) > 1.0 were defined as the cut‐off criteria. For distinguishing the consensus DEGs, volcano map analysis and heatmap analysis were used. Furthermore, the ‘VennDiagram’ package was used to select the coexisting DEGs including up‐regulated and down‐regulated genes between our data and GSE70380.

### 2.6 Construction of a prognostic model with the Least Absolute Shrinkage and Selection Operator Cox regression model

In this research, the DEGs from the Venn diagram were regarded as candidates. The prognostic classifier was established by using LASSO Cox regression analysis based on the ‘glmnet’ package. The shrinking and selecting variables were successfully achieved by the Cox regression model with the LASSO penalty. According to each prognostic genes' relative expression and its correlated coefficient, the risk score of each patient was calculated. Based on individual normalized gene expression multiplied by the LASSO Cox coefficient, a risk score formula was established, as showed below:

$$\text{Risk score}=\sum_{i=1}^{n}\left\{\text{Coefficient}\left({\text{mRNA}}_{i}\right)*\text{Expression}\left({\text{mRNA}}_{i}\right)\right\}$$

In each cohort, the cut‐off value was set to the median risk score, and these patients with risk scores higher than the median were assigned to high‐risk subgroup, while the others were assigned to low‐risk subgroup. Through using the ‘pROC’ package, the area under curve (AUC) value of the receiver operating characteristic (ROC) curve was used to test the accuracy of this prognostic model. Furthermore, the expression differences of these candidates between different clinicopathological subgroups, for example, Gleason scores (>7 vs. <=7) and pathological tumour grade (T3 + T4 vs. T1 + T2) were compared. The survival differences between low‐ and high‐risk subgroups were analysed by Kaplan–Meier (K‐M) survival analysis through the use of ‘survminer’ package and two‐way log‐rank tests. To further verify the clinical usage of this classifier, we tested it in four external databases (including MSKCC, GSE116918, GSE70769 and GSE46602).

### 2.7 Gene set enrichment analysis

According to the median risk score calculated by the classifier, the patients from the TCGA‐PRAD cohort were divided into low‐ and high‐risk subgroups. We employed the GSEA analyses to compare the difference between the two subgroups at pathway levels using ‘ggplot2’ package on R software.

### 2.8 Multivariate Cox regression analyses, nomogram ROC and subgroup analyses

Multivariate Cox regression analyses were performed to prove the five‐ERRG‐based classifier served as an indicator of RFS independent of clinicopathological features through using ‘survival’ and ‘survminer’ R packages. Hazard ratios (HRs) were determined by using the ‘Coxph’ R function. To compare the predictive values between classifiers (gene‐based classifier and clinicopathological‐feature‐based classifier), we performed the nomogram ROC analyses, which could also display the synthesizing effects by combining all the classifiers by using ‘survival’, ‘survminer’ and ‘riskRegression’ R packages. Besides, subgroup analyses were carried out by using ‘survival’ and ‘survminer’ R packages to test the application value of the five‐ERRG‐based classifier in different clinicopathological subgroups, such as pathological tumour stage (T3 + T4 vs. T1 + T2), different Gleason score (>7 vs. <= 7), age (> 60 vs. <= 60) and PSA (>10 vs. <= 10) subgroups.

### 2.9 Function verification of TK1 at the cellular level

The function of UBE2T, HERPUD1, IQGAP3 and IGFBP3 had been demonstrated in Pca, while the roles of TK1 have rarely been investigated, so we verified the function of TK1 at the cellular level. The shRNA duplex sequences (shTK1: forward 5'‐TGTCGGCTCTGCTACTTCAAG‐3′, reverse 5'‐CTTGAAGTAGCAGAGCCGACA‐3′) and the scrambled control (scRNA) were used to silence TK1. C4‐2R and 22Rv1 cells were plated into 6 cm culture dishes and were transfected with 50 nmol/L of the shRNA or scRNA when the density of cells reached with 60%–70%. After 24 h, shRNAs or scRNAs were removed and cells were incubated with culture medium.

5000 cells of C4‐2R and 22Rv1 were plated into each well of 24‐well plates. Following with MTT assays, we harvested cells on Day 0, 2, 4 and 6. Added 50 μl of 5 mg/ml MTT into each well and the plates were incubated at 37°C with 5% CO_2_ for 2 h. After the incubating, media was removed and 1 ml DMSO was added into each well to dissolve the precipitate. The plates were covered with foil and be placed on an orbital shaker for 20 min, after that, the absorbance of each well was measured at 570 nm. 1000 cells of C4‐2R and 22Rv1 were plated into 6 cm culture dishes. After two‐week Enzalutamide treatment, the colonies were fixed with methanol and treated with crystal violet solution. The nonoverlapping group of at least 50 cells was defined as a colony.

### 2.10 Western blotting

Our previous studies had described the detailed procedures of the Western blotting.^23^ In short, RIPA lysis buffer was used to lyse C4‐2R or 22Rv1 to extract the total proteins, and the BCA protein assay kit was used to determine the protein concentration. 12.5% SDS‐polyacrylamide gels were used to separate the samples followed by transferring onto NC membranes (Bio‐Rad, Hercules, CA). 5% nonfat milk was used to block the membranes at room temperature for 1 h, following by primary antibodies and the appropriate secondary antibodies (1:5000; goat anti‐rabbit; Proteintech Group, No. SA00004‐2) were used to incubate the membranes in order.

### 2.11 IHC validation

The study was approved by the Ethics committee of The First Affiliated Hospital of Anhui Medical University. Rabbit anti‐TK1 which was purchased from Bioss Antibodies (Bioss Antibodies, Peking, China) was used for immunohistochemistry (IHC) of TK1 expression in tissue samples. The staining intensity was divided into three levels: 0, negative; 1, weak; 2, medium; and 3, strong. The degree of staining was divided into four levels: 0, 0%; 1, 1%–25%; 2, 26%–50%; 3, 51%–75%; and 4, > 76%. The final score was obtained by the sum of the intensity score and the quantity score. Scores of ≥2 were defined as a positive expression, while others were defined as a negative expression.^24^

### 2.12 Statistical analysis

R software 3.6.1 (MathSoft, Cambridge, MA) was used to perform the statistical analyses. Student's t‐tests (two‐tailed unpaired) were performed by using GraphPad Prism software (GraphPad Software, San Diego, CA) to evaluate the functional differences after silencing the expression of critical candidates. In this study, the *p* value <0.05 was considered statistically significant.

## 3 RESULTS

### 3.1 Identification of Enzalutamide‐resistance‐related‐genes

**FIGURE 1:**
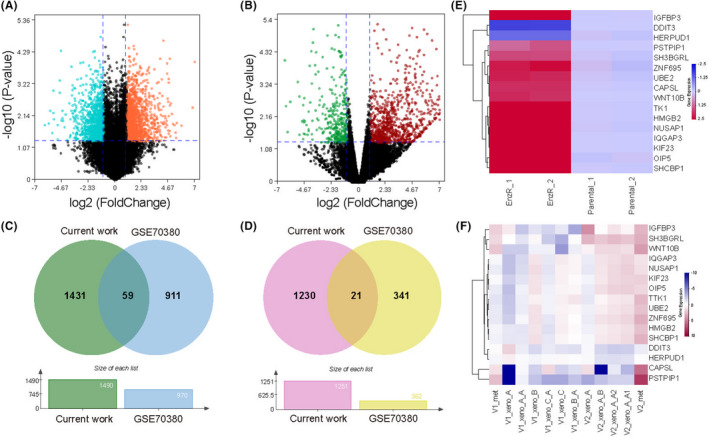
Identification of Enzalutamide‐resistance‐related‐genes (ERRGs). **A** The ERRGs between C4‐2 and C4‐2R in our current work. **B** The ERRGs between pre‐ and post‐Enzalutamide treatment based on patient‐derived Xenograft (PDX) models in GSE70380. **C** The up‐regulated ERRGs in our current work and GSE70380. **D** The down‐regulated ERRGs in our current work and GSE70380. **E** The expressions of 16 prognostic ERRGs between C4‐2 and C4‐2R in our current work. **F** The expressions of 16 prognostic ERRGs between pre‐ and post‐Enzalutamide treatment based on patient‐derived Xenograft (PDX) models in GSE70380

We extracted the total RNA from the C4‐2 and C4‐2R cells. Then, we performed the microarrays to detect the expression of genes from the extracted total RNA, with annotated row read genes of 22,586 genes. For those genes, the adjusted *p*‐value < 0.05 and absolute log_2_ (fold change) > 1.0 were defined as statistically significant ERRGs. Volcano map was used to display the DEGs in two datasets(Figure 1A‐B). For our data, we found that the up‐regulated and down‐regulated genes between C4‐2 and C4‐2R cells were 1490 and 1251, respectively. For the RNA‐seq of GSE70380, we found that the up‐regulated and down‐regulated genes were 970 and 362, respectively. We overlapped the ERRGs from the two datasets to obtain more convincing ERRGs. Through overlapping comparison of ERRGs from the two datasets, we found that up‐regulated ERRGs and down‐regulated ERRGs were 59 and 21, respectively (Figure 1C‐D). For the total 80 ERRGs, we evaluated their prognostic value in TCGA‐PRAD cohort, and only 16 DEGs were retained with the statistical significance of prognostic value via univariate Cox regression analysis. The heatmap was used to illustrate the 16 ERRGs expression profiles of our dataset and GSE70380 (Figure 1E‐F).

### 3.2 Identification of prognostic genes in predicting the RFS of Pca patients

**FIGURE 2:**
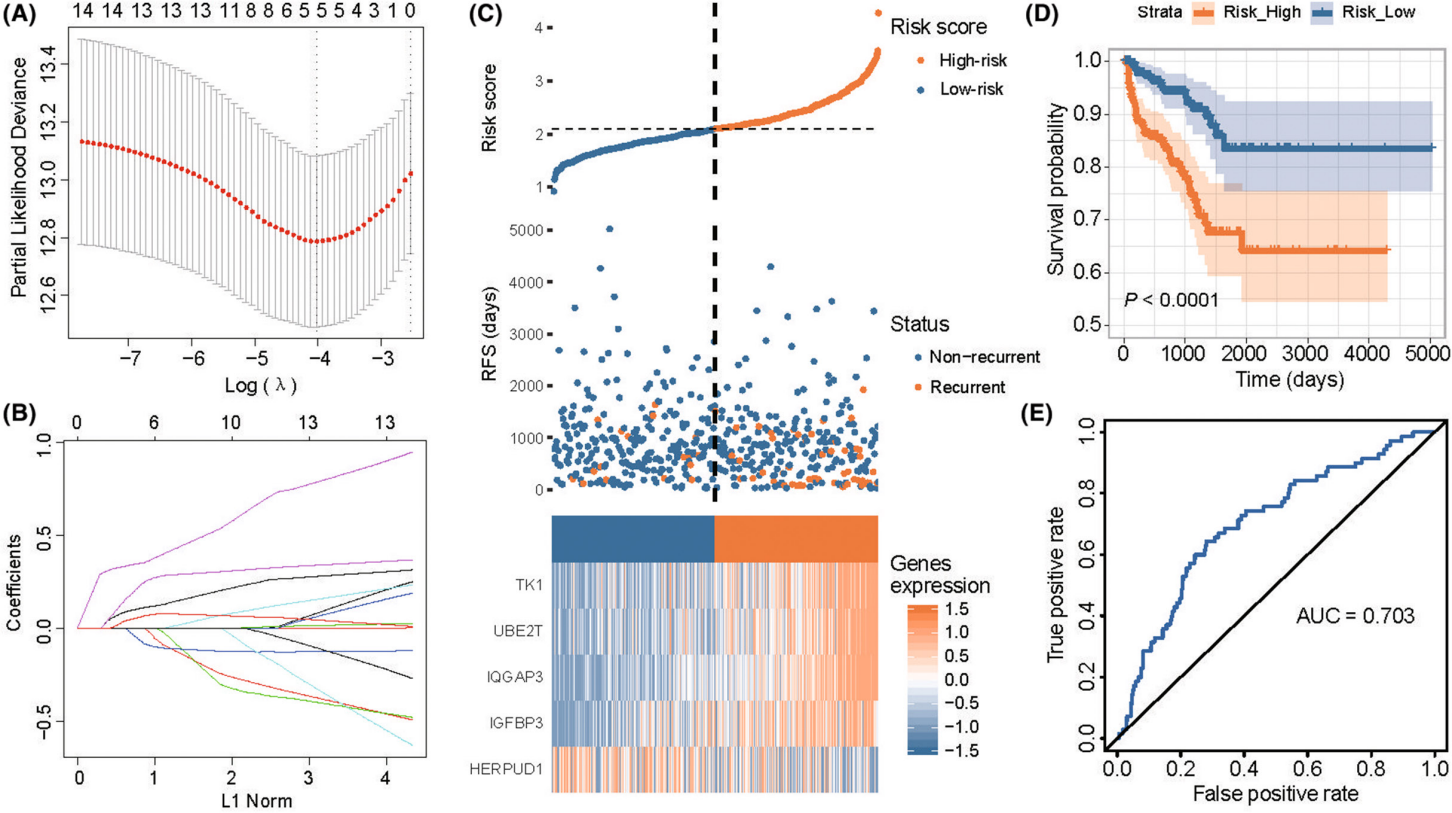
Identification of prognostic genes in predicting the RFS of PCa patients in training cohort. **A** The optimal parameter (lambda) selection in LASSO model using ten‐fold cross‐validation via minimum criteria. **B** LASSO coefficient profiles of the selected features. **C** The distribution of risk scores and the survival status of PCa patients and the expression heatmap of five prognostic ERRGs in training cohort. **D** The Kaplan–Meier analysis on the training cohort. **E** The predictive value of the five‐ERRG‐based classifier in the training cohort

Then, based on the 16 genes obtained from univariate Cox analysis, the key genes with the strongest RFS predictive ability were performed by using LASSO Cox regression analysis. Five genes were extracted in this process, such as UBE2T, HERPUD1, IQGAP3, IGFBP3 and TK1(Figure 2A‐B). K‐M analysis was performed to examine the relationship between each gene of those five DEGs and the RFS of Pca patients. We found that the high expression of HERPUD1 (Figure S1A: *p* = 0.011 for HERPUD1) acted as a protective factor in Pca patients and the high expression of UBE2T, IQGAP3, IGFBP3 and TK1 indicated unfavourable prognoses of Pca patents in the TCGA cohort (Figure S1B‐E: *p* = 0.00014 for IGFBP3, *p* < 0.0001 for TK1, *p* < 0.0001 for UBE2T and *p* < 0.0001 for IQGAP3). Furthermore, we compared the expression differences of five consensus genes by the dichotomized subgroups, such as T stage and Gleason score (Figure S1F‐G). We found that low expression of HERPUD1 and the high expression of UBE2T, IQGAP3, IGFBP3 and TK1 were observed in the T stage (*p* < 0.05) and Gleason score (*p* < 0.05) subgroups. In view of those genes, we built a five‐gene‐based classifier. Calculating the classifier risk score of each patient based on the sum of 5 genes expression multiplied by the coefficient: the risk score = 0.106 × expression (UBE2T) – 0.060 × expression (HERPUD1) + 0.349 × expression (IQGAP3) + 0.222 × expression (IGFBP3) + 0.059 × expression (TK1). The TCGA data was defined as the training cohort. According to this classifier, Pca patients were divided into high‐ and low‐risk groups. Patients with low‐risk scores indicated better survival outcomes when compared with patients with high‐risk scores (Figure 2C‐D *p* < 0.0001). Besides, the ROC curve proved the sensitivity and stability of this model. The model obtained a satisfactory prediction in the training cohort (Figure 2E, AUC = 0.703).

### 3.3 Verification of prognostic genes in predicting the RFS of Pca patients in our current work

**FIGURE 3:**
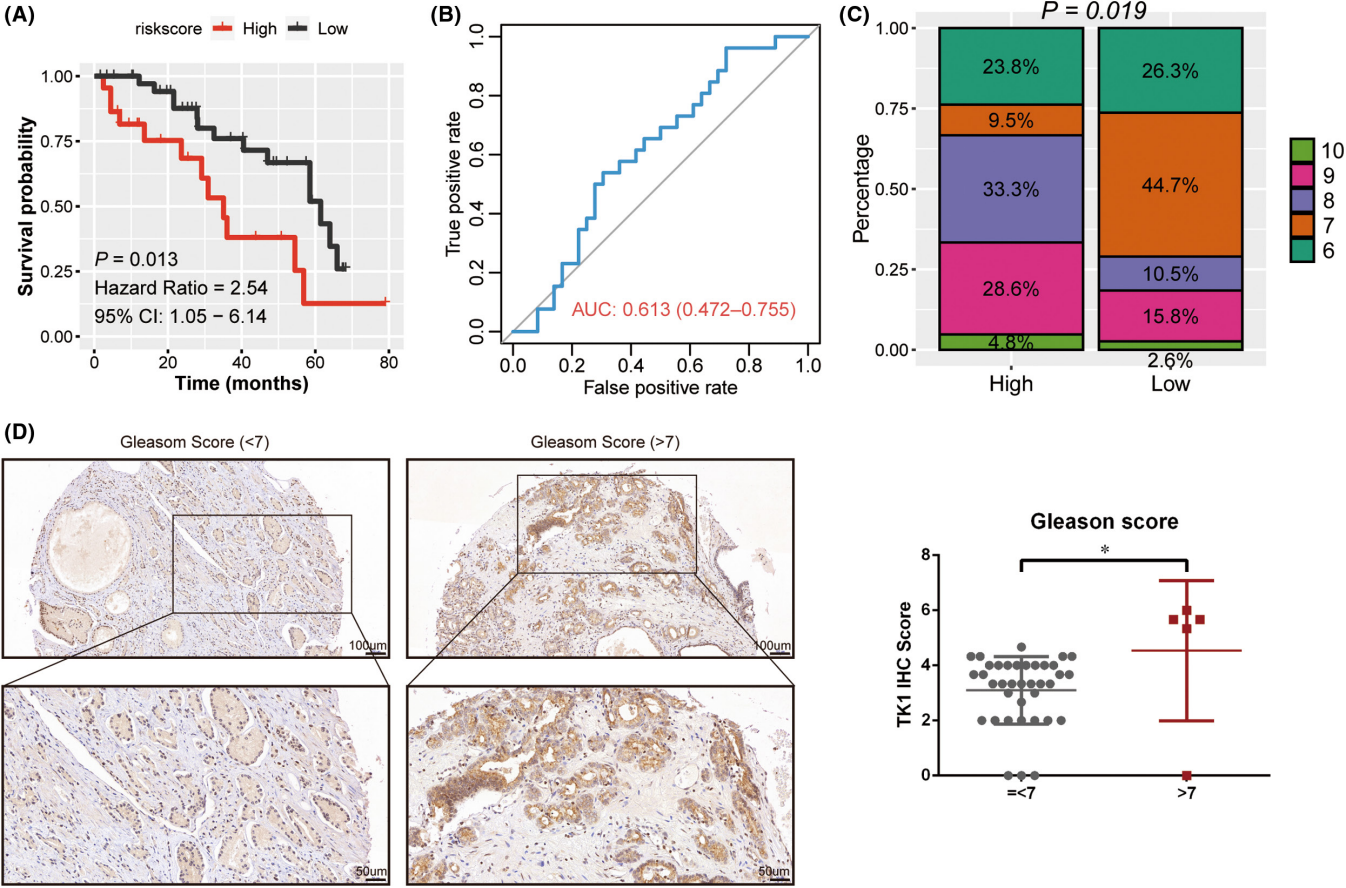
Verification of prognostic genes in predicting the RFS of PCa patients in our current work. **A** The Kaplan–Meier analysis on our current work. **B** The predictive value of the five‐ERRG‐based classifier in our current work. **C** The distribution of Gleason score of high‐ or low‐risk group. **D** The intensity and degree of staining for the IHC of TK1 for prostate tissue in patients with Gleason score = <7 or >7. The results are represented as mean ± standard derivation. *p < 0.05 vs. Gleason score = <7

Each sample's risk score from our owe AHMU‐PC cohort was calculated based on the above‐mentioned formula to further validate the prognostic value of 5 ERRGs. K‐M survival curves confirmed that the RFS of patients in low‐risk group was significantly longer than those in high‐risk group (Figure 3A: HR = 2.54, 95%CI: 1.05–6.14, *p* = 0.013) and the nomogram ROC curve demonstrated that the nomogram had an increased predictive values (Figure 3B: AUC = 0.613, 95%CI: 0.472–0.755). In the high‐risk group, 66.7% of patients had a Gleason score higher than 7, while 71% of the patients in the low‐risk group had a Gleason score less than or equal to 7 (Figure 3C). In addition, we performed IHC staining of TK1 on prostate tissues of Pca patients. In Table 1, We found that the high expression of TK1 was observed in the envelope invasion (*p* = 0.01), Gleason score (*p* = 0.044) and pathology stage (*p* = 0.02) subgroups. The IHC scores of specimens with Gleason score higher than 7 were significantly higher than that of specimens with Gleason score less than or equal to 7 (Figure 3D: *p* < 0.05).

**TABLE 1:** Association between TK1 protein level and pathological features in tissue array

Parameter	IHC results for TK1			
	Strong Positive (*n*, %)	Weak Positive (*n*, %)	Negative (*n*, %)	*p* value
Age				0.086
< 60	1	3	2	
≥ 60	15	19	2	
Envelope invasion				0.01*
No	11	22	4	
Yes	5	0	0	
Seminal vesicle invasion				0.435
No	15	22	4	
Yes	1	0	0	
Gleason score				0.044*
≤ 7	12	22	3	
> 7	4	0	1	
Pathology Stage				0.02*
I‐II	11	22	3	
III‐IV	5	0	1	

* , *p* < 0.05.

### 3.4 Exploring the biological function of TK1 in C4‐2R and 22Rv1

**FIGURE 4:**
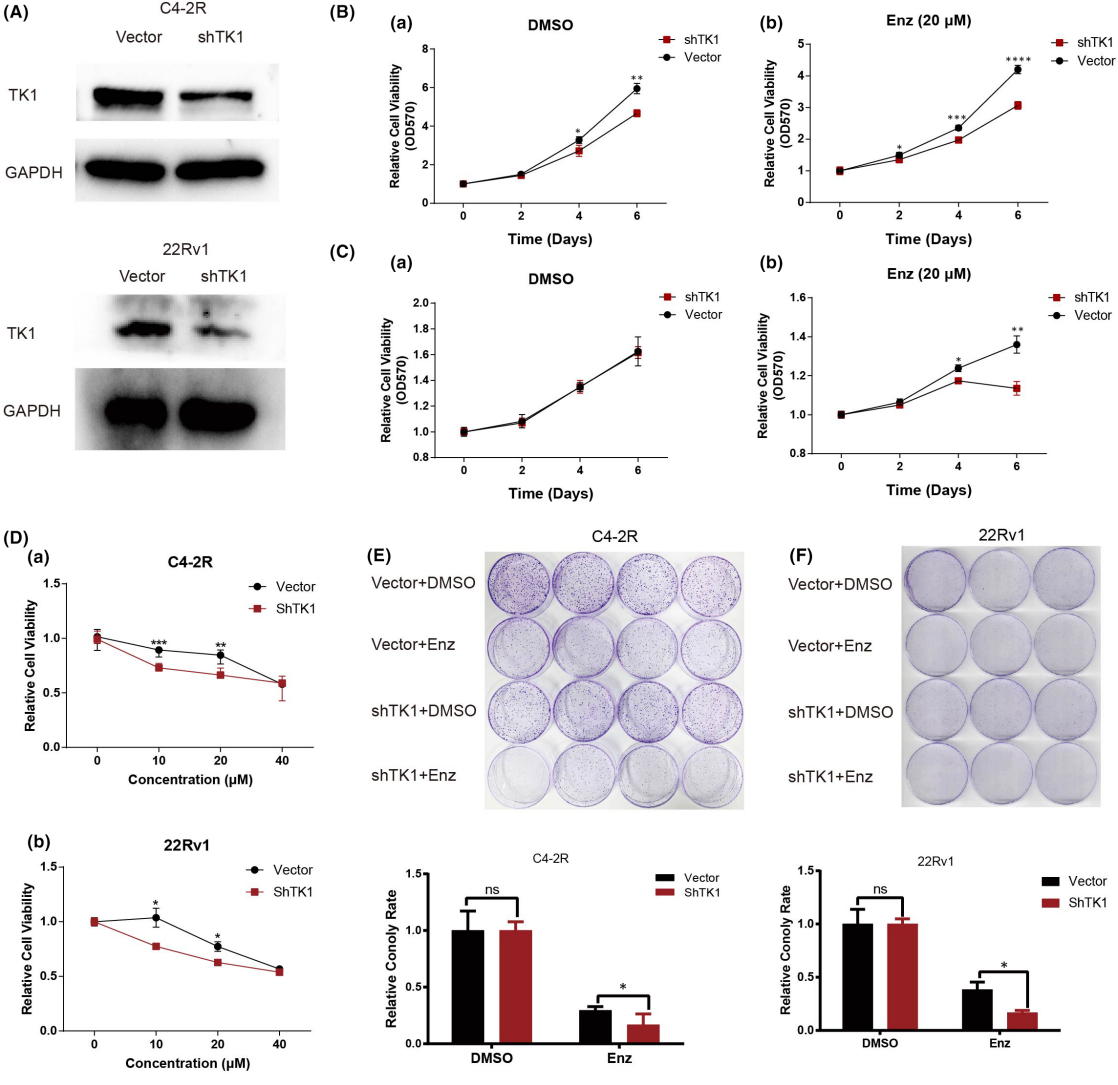
Exploring the biological function of TK1 in C4‐2R and 22Rv1. **A** The protein levels of TK1 in C4‐2R and 22Rv1 after the transfection of shTK1. **B, C** The proliferative ability of C4‐2R (**B**) and 22Rv1 (**C**) was remarkably reduced after the treatment with enzalutamide by TK1‐knockdown. **D** The proliferative ability of C4‐2R (**a**) and 22Rv1 (**b**) was remarkably reduced after the treatment with different concentrations of enzalutamide by TK1‐knockdown. **E, F** Knockdown of TK1 inhibits cell proliferation in C4‐2R (**E**) and 22Rv1 (**F**) after the treatment with enzalutamide assessing by the colony formation assay. Data are summary of 3 independent experiments. * *p* < 0.05, ** *p* < 0.01, ***p < 0.005 vs. scRNA‐transfected cells

Short hairpin RNA (shRNA) was used to explore the biological function of TK1 in Pca cell lines by transfecting C4‐2R and 22Rv1, which were resistant to Enzalutamide. After C4‐2R and 22Rv1 were transferred with shTK1 for 24 h, the expression levels of TK1 in C4‐2R and 22Rv1 were detected by WB. The results of WB showed that shTK1 could significantly decreased the expression of TK1 in C4‐2R and 22Rv1 (Figure 4A). MTT experiments demonstrated that the proliferative ability of C4‐2R and 22Rv1 was remarkably reduced by TK1‐knockdown (Figure 4B‐D). The colony formation experiments revealed that the number of colonies in the shTK1 transfection group was remarkably decreased compared with that in the scRNA transfection group (Figure 4E‐F). These results indicated that knowndown of TK1 significantly reduced the progression of C4‐2R and 22Rv1.

### 3.5 Significance and stability assessment of the five‐gene‐based RFS predicting classifier in external validation cohorts

**FIGURE 5:**
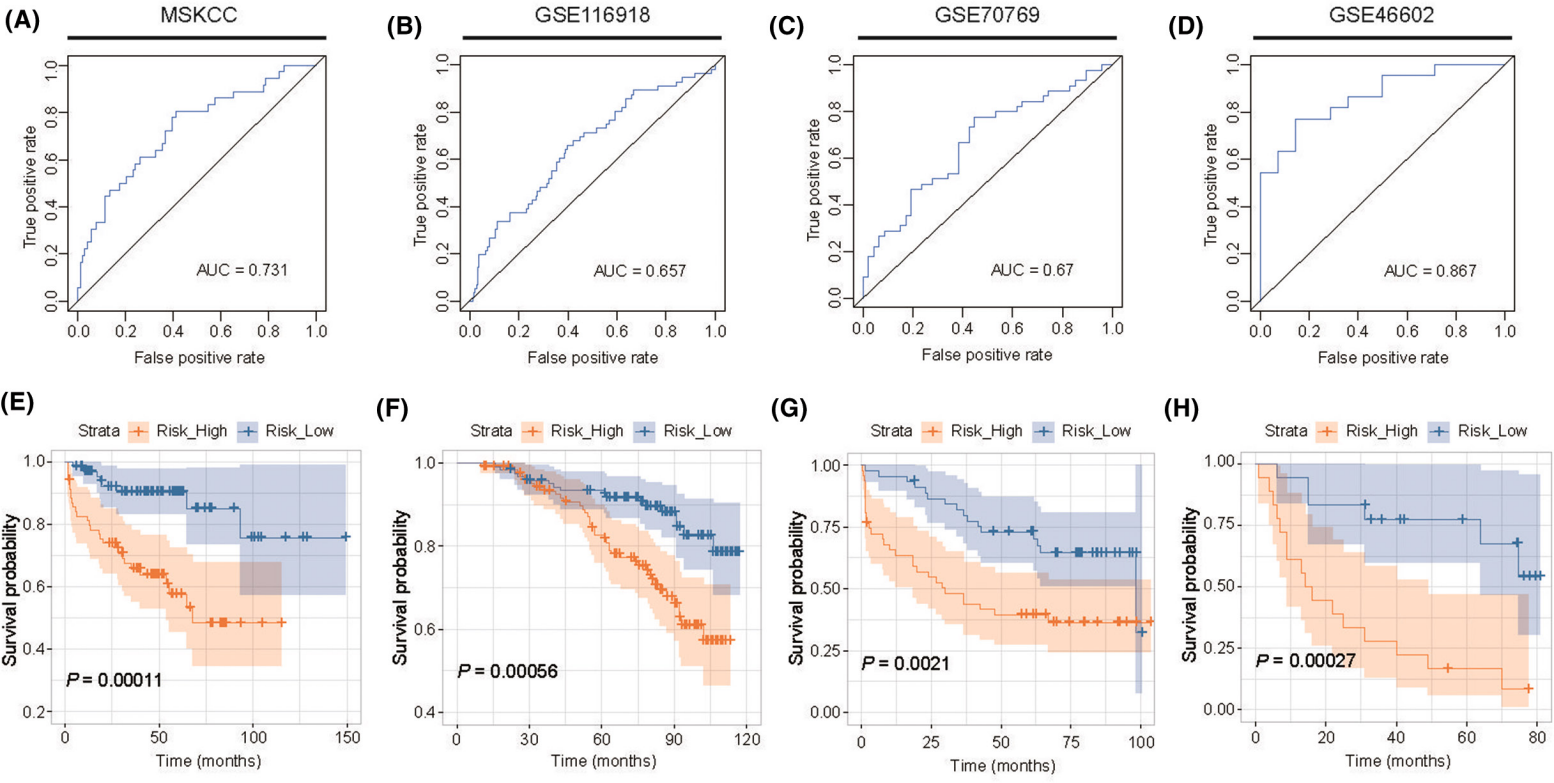
Significance and stability assessment of the five‐gene‐based RFS predicting classifier in validation cohorts. **A‐D** The Kaplan–Meier analysis on the validation cohorts of MSKCC (**A**), GSE116918 (**B**), GSE70769 (**C**) and GSE46602 (**D**). **E‐H** The predictive value of the five‐ERRG‐based classifier in validation cohorts of MSKCC (**E**), GSE116918 (**F**), GSE70769 (G) and GSE46602 (**H**)

In order to further verify the accuracy and stability of this model, we selected four external databases (MSKCC, GSE116918, GSE70769 and GSE46602) as validation cohorts. The ROC curves of four validation cohorts showed that this model had the good predictive ability (Figure 5A‐D, AUC = 0.731 in MSKCC cohort, AUC = 0.657 in GSE116918 cohort, AUC = 0.670 in GSE70769 cohort and AUC = 0.867 in GSE46602 cohort). The K‐M analyses of four validation cohorts showed that the patients in high‐risk groups had worse survival outcomes than those in low‐risk groups (Figure 5E‐H *p* = 0.00011 in MSKCC cohort, *p* = 0.00056 in GSE116918 cohort, *p* = 0.0021 in GSE70769 cohort and *p* = 0.00027 in GSE46602 cohort).

### 3.6 Significant signalling pathway obtained by GSEA

**FIGURE 6:**
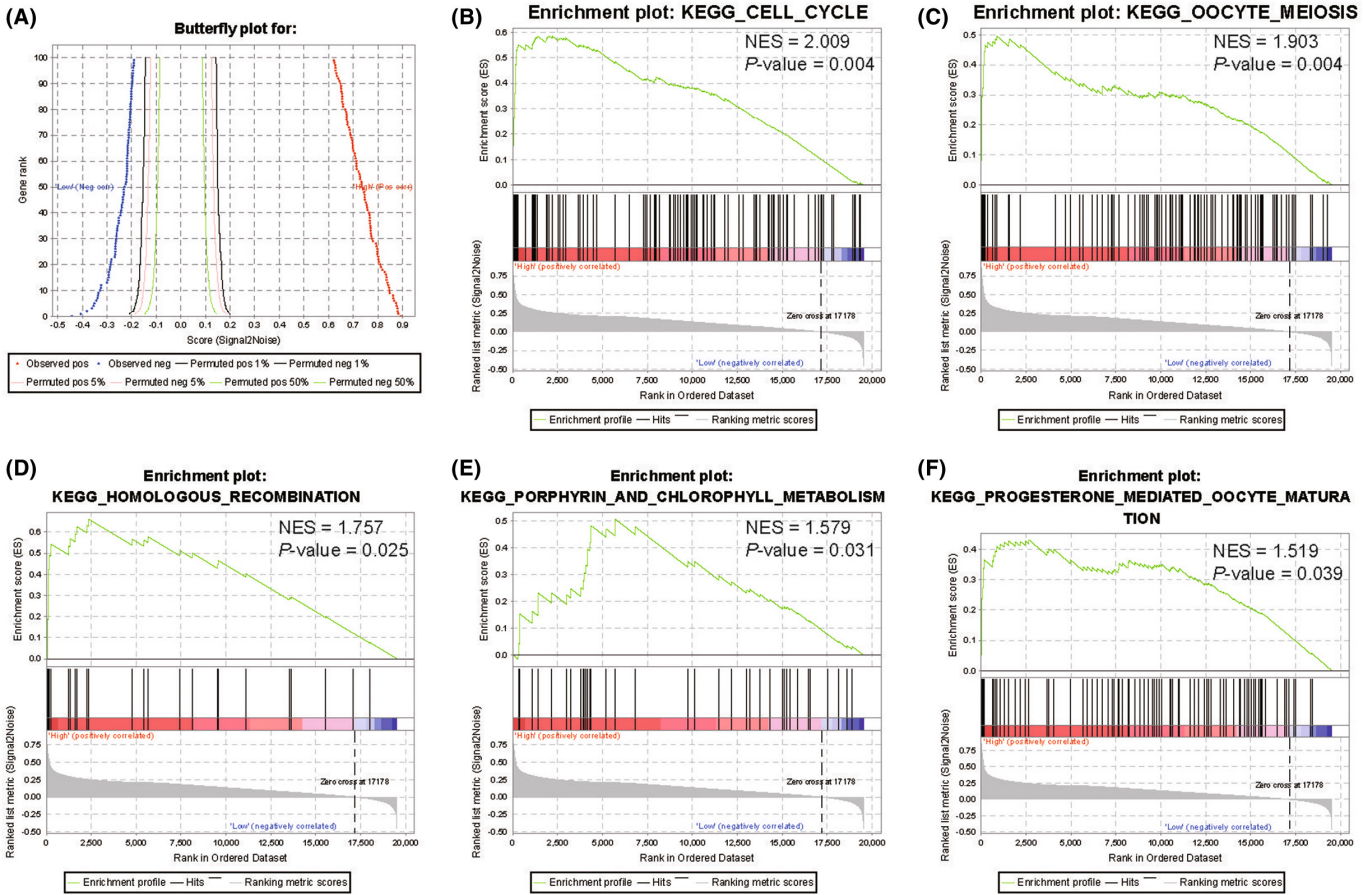
Significantly different signalling pathway between high‐ and low‐risk subgroups were obtained by GSEA

Based on the KEGG pathway database, potential signalling pathways that might play a vital role in regulating the prognosis of patients were predicted by GSEA analysis in view of the risk value of this model. The signalling pathways such as cell cycle (Normalized Enrichment Score [NES] = 2.009, *p* = 0.004), oocyte meiosis (NES = 1.903, *p* = 0.004), homologous recombination (NES = 1.757, *p* = 0.025), porphyrin and chlorophyll metabolism (NES = 1.579, *p* = 0.031) and progesterone‐mediated oocyte mature (NES = 1.519, *p* = 0.039) were enriched (Figure 6).

### 3.7 Subgroup analyses, multivariate Cox regression and nomogram ROC

Through using K‐M analyses, the five‐gene‐classifier precisely discriminated the high‐ and low‐risk groups of patients in these subgroups, such as different age, Gleason score, PSA and tumour stage subgroups (Figure S2).

In order to get the independence between the five‐gene‐cluster with clinicopathological features, multivariate Cox regression analyses were performed. The five‐gene‐classifier was serving as an independent indicator for RFS (Figure S3A‐E, TCGA cohort: HR = 1.8, 95%CI: 1.01–3.3, *p* = 0.047; MSKCC cohort: HR = 3.27, 95%CI: 1.44–7.4, *p* = 0.004; GSE116918 cohort: HR = 2.46, 95%CI: 1.35–4.5, *p* = 0.003; GSE44602 cohort: HR = 3.18, 95%CI: 1.09–9.3, *p* = 0.035; GSE70769 cohort: HR = 1.5, 95%CI: 0.81–2.9, *p* = 0.185). For the purpose of evaluating the accuracy of the five‐gene‐classifier in each cohort and clinicopathological features, the nomogram ROC curve was performed. The results showed that the nomogram had an increased predictive values in TCGA (AUC = 76.7, 95%CI: 70.4–83.0), MSKCC (AUC = 88.1, 95%CI: 78.6–97.6), GSE116918 (AUC = 70.8, 95%CI: 61.2–80.4), GSE44602 (AUC = 89.7, 95%CI: 79.4–100.0) and GSE70769 (AUC = 78.7, 95%CI: 68.9–88.5) cohorts (Figure S3F‐J).

## 4 DISCUSSION

In the currently study, based on 80 overlapped DEGs identified by two mRNA expression microarray, we established a five‐gene‐based classifier in the TCGA cohort and validated it in four independent validation cohorts, as well as our cohort data. Therefore, it has been proved that the five‐gene‐based classifier has a better prognostic value for RFS in PCa patients than age, Gleason score and PSA values, and has a similar prognostic value with pathological T stage. Following the advancement of molecular biology, the arrival of the era of big data has become a reality. Researchers tried to use the large amounts of data to stratify risk and optimize chemotherapy strategies for various tumour types.^25^, ^26^, ^27^

So far, the relationship between prognosis of PCa and differential expression of certain genes had been revealed by only a few studies.^28^, ^29^ In addition, large‐scale, independent validation of the genes in these researches has not been undergo. In this study, the good prognostic value of the five‐gene‐based classifier for predicting the recurrence risk of PCa has been showed according to the digital expression profiling, which has been validated in the external cohorts and our data. Notably, we also validated the classifier in our cohort, which comprising the FFPE samples and obtained consistent results. All these results proved the feasible and applicable model in predicting the RFS of PCa patients.

Drug treatment resistance is a complicated process, the exploration of dysregulated genes related to the resistance and progression of PCa will help provide more therapeutic options and further improve prognosis. In the current study, a set of five genes was identified with the ability to predict clinical outcomes effectively. HERPUD1, as an endoplasmic reticulum‐bound protein, is a putative component of the endoplasmic reticulum‐associated protein degradation (ERAD) pathway.^30^ The loss of HERPUD1 can increase the sensitivity of cells to endoplasmic reticulum stress and apoptosis.^31^ Recently, study showed that the lower mRNA expression of HERPUD1 was associated with a higher incidence of metastasis after radical prostatectomy.^32^ IGFBP3 as a multifunctional protein plays a key role in the regulation of IGF I/II activity, cell proliferation and apoptosis. Recent research found that overexpression of IGFBP3 was associated with tumour recurrence and poor patient survival through analysis of human prostate cancer specimens and TCGA patient cohorts.^33^ As a member of the E2 family, UBE2T is responsible for the ATP‐dependent ubiquitin labelling of target proteins to promo their degradation. Some studies showed that the overexpression of UBE2T could promote the proliferation of breast cancer cells by repressing BRCA1 expression.^34^ Recently studies found that the high expression level of UBE2T was related to tumour formation, invasion, metastasis and poor disease‐free survival of PCa patients.^35^ Thymidine kinase 1 (TK1) participates in the synthesis of DNA precursor and acts as a biomarker for cancer including prostate and breast cancer,^36^ but the specific role of TK1 in the occurrence and development of PCa is unclear. IQGAP3, as a member of the IQGAP family, promotes the proliferation, migration and invasiveness of cancer cells through interacting with its target proteins.^37^ Some researches had found that the expression level of IQGAP3 in PCa was increased and the expression level of IQGAP3 correlated inversely with survivability,^37^ but the specific role of IQGAP3 in the occurrence and development of PCa is unclear.

Further studies of gene functions and pathways particularly cell cycle, oocyte meiosis, homologous recombination, porphyrin and chlorophyll metabolism, and progesterone‐mediated oocyte maturation are helpful to understand the pathophysiologic mechanism of Enzalutamide resistance in PCa (Figure 6). The cell cycle is a complex process that guides cell proliferation through a series of checkpoints that can correct DNA damage, genetic abnormalities and other errors.^38^ In most of the human cancer, the G1 progression of cell cycle is out of control and the impairing of cell cycle checkpoints results in the accumulation of genetic aberrations.^39^ In the level of immunology, the cancer cells and egg cells have the same behaviour: implement and development. A study found that as reprogrammed cells, cancer cells have the initiation of the egg cells' genetic program.^40^ In human cancers, the activation of meiosis can drive the oncogenesis of cancer.^41^ For restarting stalled replication forks, repairing spontaneous DNA double‐strand breaks and generating genetic diversity, homologous recombination (HR) is an essential pathway. The tumour cells which were defective in HR were more sensitive to many anticancer drugs. Many studies had found that the re‐expression of HR in tumour cells may be one of the reasons for the drug resistance.^42^

As far as we know, this is the first attempt to use Enzalutamide treatment resistant‐related genes to set up a classifier for RFS prediction. Our five‐gene‐based classifier is a useful prognostic biomarker for patients with PCa. A nomogram comprised of the five‐gene‐based classifier and clinicopathological variables may contribute to individual prognosis prediction and treatment decision‐making.

## AUTHOR CONTRIBUTIONS

**Jing Chen:** Data curation (equal); project administration (equal); writing – original draft (equal). **Jialin Meng:** Investigation (equal); writing – review and editing (equal). **Yi Liu:** Project administration (equal); validation (equal). **Zichen Bian:** Investigation (equal); resources (equal). **Qingsong Niu:** Investigation (equal); resources (equal). **Junyi Chen:** Validation (equal). **Jun Zhou:** Data curation (equal); investigation (equal). **Li Zhang:** Conceptualization (equal); data curation (equal). **Meng Zhang:** Formal analysis (equal); supervision (equal); writing – review and editing (equal). **Chaozhao Liang:** Conceptualization (equal); funding acquisition (equal); writing – review and editing (equal).

## FUNDING INFORMATION

The National Natural Science Foundation of China 81802827 and 81630019. Scientific Research Foundation of the Institute for Translational Medicine of Anhui Province (2017ZHYX02). The Natural Science Foundation of Guangdong Province, China (2017A030313800).

## CONFLICT OF INTEREST

None.

## Supporting information


**Figure S1** The relationship between the five ERRGs and prognosis of PCa patients. A The high expression of HERPUD1 was linked with a favourable prognosis. (B‐E) Highly expressed IGFBP3 (B), TK1 (C), UBE2T (D) and IQGAP3 (E) predicted a poor prognosis. The expression levels of those five ERRGs different subgroups of stage (**F**) and Gleason score (**G**).

**Figure S2** Stratified analyses for different clinicopathological subgroups in the TCGA, MSKCC, GSE116918, GSE44602 and GSE70769.

**Figure S3** Validation of the prognostic signature of the five‐ERRG‐based classifier. A‐E The hazard ratio of each independent risk factor to the five‐ERRG‐based classifier in the validation cohorts of TCGA (A), MSKCC(B), GSE116918 (C), GSE46602 (D) and GSE70769 (E). Nomogram ROC analyses by synthesizing the five‐ERRG‐based classifier and clinicopathological features in the validation cohorts of TCGA (F), MSKCC(G), GSE116918 (H), GSE46602 (I) and GSE70769 (J). *p < 0.05, **p < 0.01, ***p < 0.005 vs. low‐risk groups.

## Data Availability

Data available on request from the authors: The data that support the findings of this study are available from the corresponding author upon reasonable request.
