# Supplementary figures and images for: Establishment of a five‐enzalutamide‐resistance‐related‐gene‐based classifier for recurrence‐free survival predicting of prostate cancer
Source: J Cell Mol Med. 2022 Sep 28;26(21):5379–90. doi: 10.1111/jcmm.17554 (PMC9639034; doi:10.1111/jcmm.17554)

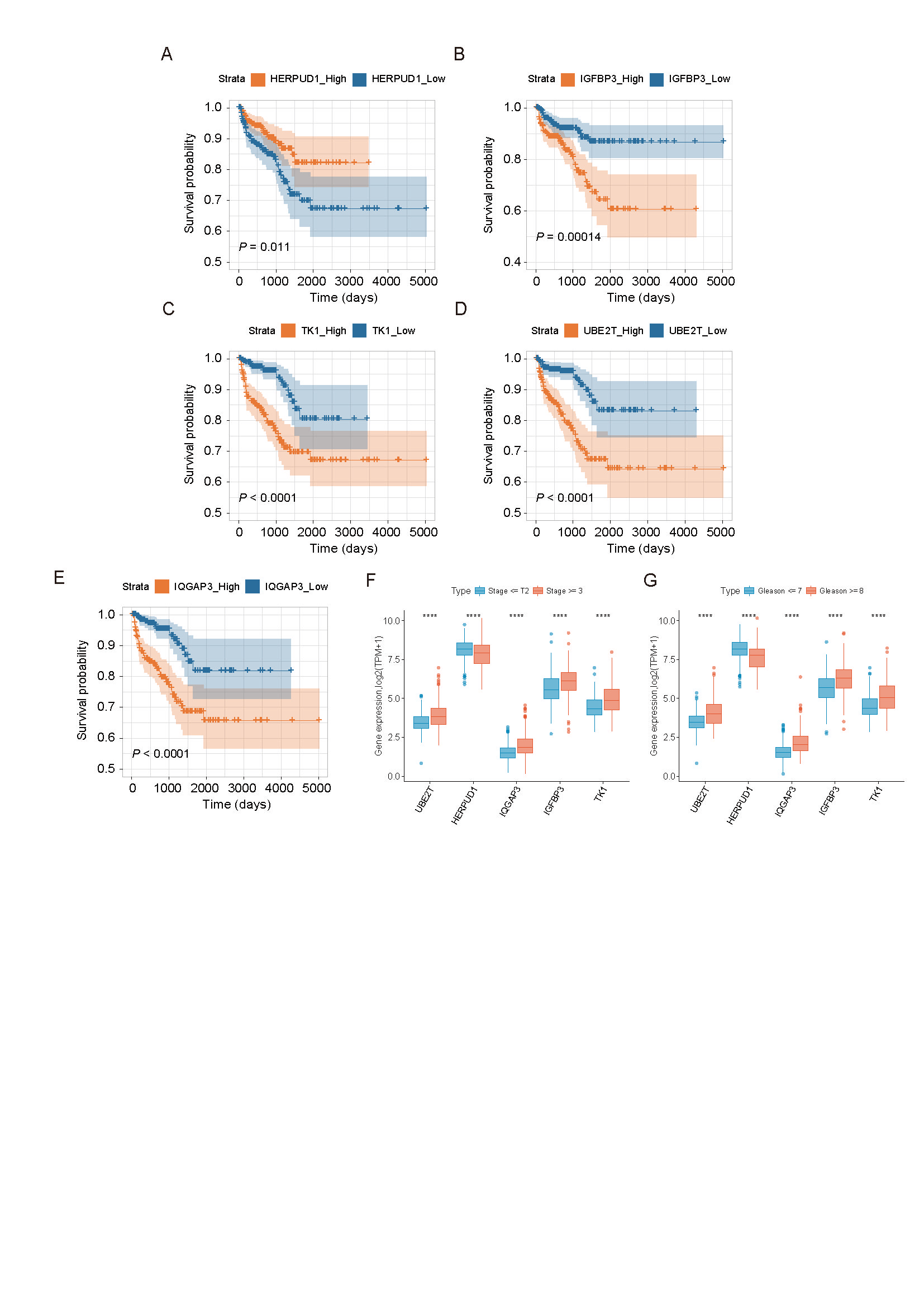

Supplement: Supplementary file 1 — Figure S1 The relationship between the five ERRGs and prognosis of PCa patients. A The high expression of HERPUD1 was linked with a favourable prognosis. (B‐E) Highly expressed IGFBP3 (B), TK1 (C), UBE2T (D) and IQGAP3 (E) predicted a poor prognosis. The expression levels of those five ERRGs different subgroups of stage (F) and Gleason score (G). [file JCMM-26-5379-s003.tiff]

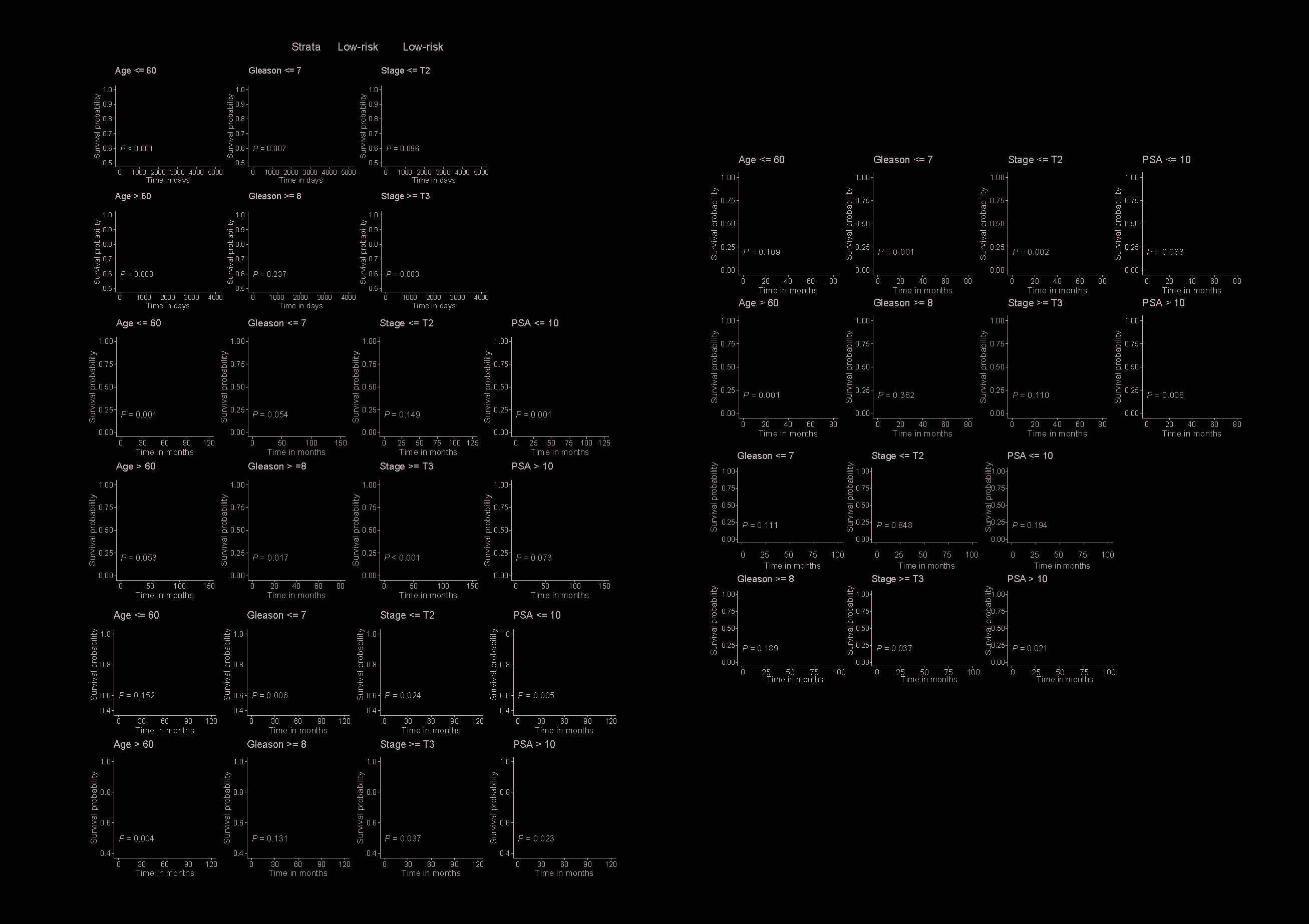

Supplement: Supplementary file 2 — Figure S2 Stratified analyses for different clinicopathological subgroups in the TCGA, MSKCC, GSE116918, GSE44602 and GSE70769. [file JCMM-26-5379-s002.tiff]

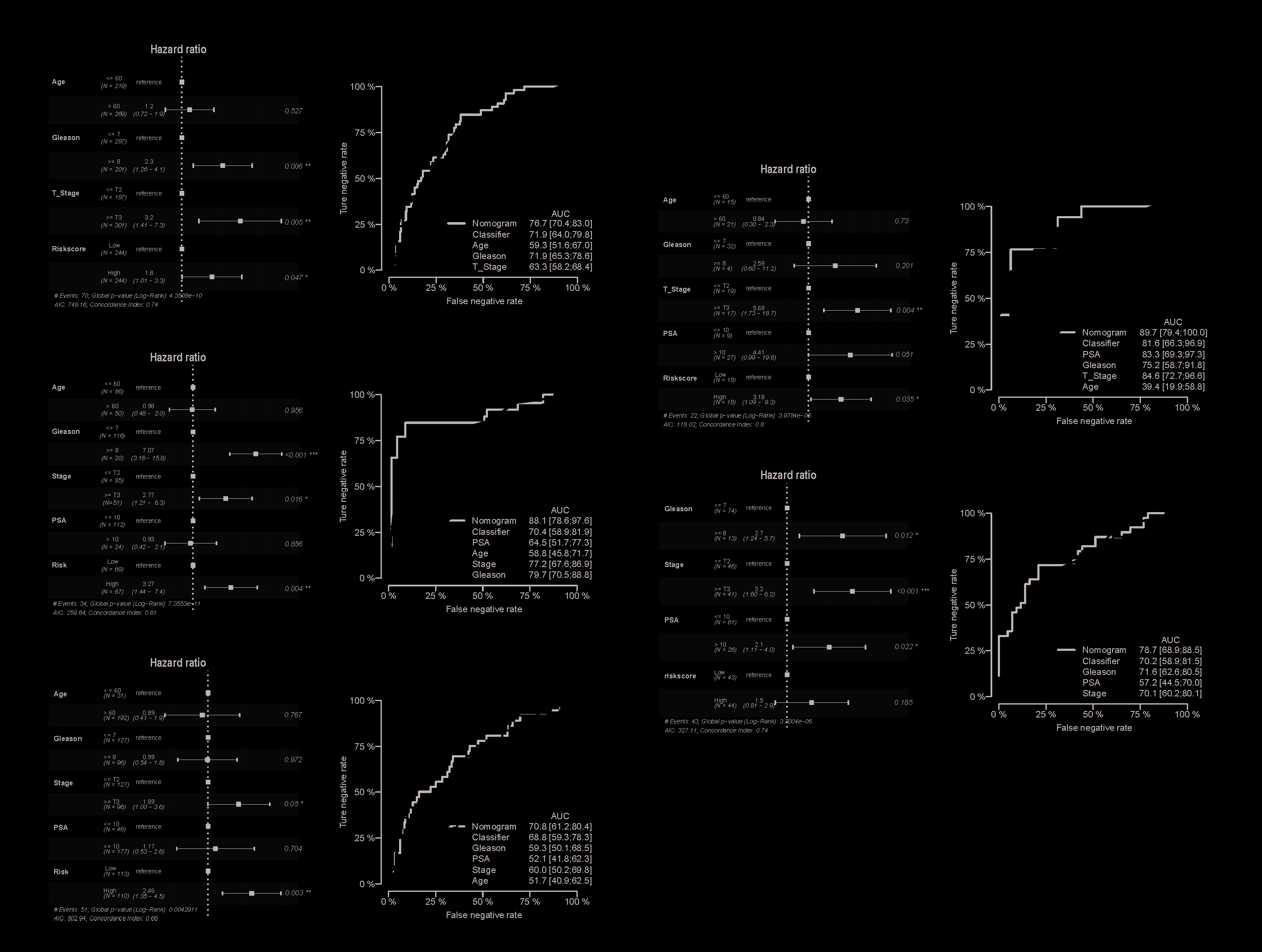

Supplement: Supplementary file 3 — Figure S3 Validation of the prognostic signature of the five‐ERRG‐based classifier. A‐E The hazard ratio of each independent risk factor to the five‐ERRG‐based classifier in the validation cohorts of TCGA (A), MSKCC(B), GSE116918 (C), GSE46602 (D) and GSE70769 (E). Nomogram ROC analyses by synthesizing the five‐ERRG‐based classifier and clinicopathological features in the validation cohorts of TCGA (F), MSKCC(G), GSE116918 (H), GSE46602 (I) and GSE70769 (J). *p < 0.05, **p < 0.01, ***p < 0.005 vs. low‐risk groups. [file JCMM-26-5379-s001.tiff]
